# Gastrointestinal Disorders and Metabolic Syndrome: Dysbiosis as a Key Link and Common Bioactive Dietary Components Useful for their Treatment

**DOI:** 10.3390/ijms21144929

**Published:** 2020-07-13

**Authors:** Anna De Filippis, Hammad Ullah, Alessandra Baldi, Marco Dacrema, Cristina Esposito, Emanuele Ugo Garzarella, Cristina Santarcangelo, Ariyawan Tantipongpiradet, Maria Daglia

**Affiliations:** 1 Department of Pharmacy, University of Naples Federico II, 80131 Naples, Italy; anna.defilippis@unina.it (A.D.F.); hammad.ullah@unina.it (H.U.); marco.dacrema@unina.it (M.D.); cristina.esposito@unina.it (C.E.); emanueleugo.garzarella@unina.it (E.U.G.); cristina.santarcangelo@unina.it (C.S.); ariyawanps@gmail.com (A.T.); 2 TefarcoInnova, National Inter-University Consortium of Innovative Pharmaceutical Technologies—Parma, 43124 Parma, Italy; alessandra.baldi.alimenti@gmail.com; 3 International Research Center for Food Nutrition and Safety, Jiangsu University, Zhenjiang 212013, China

**Keywords:** gastrointestinal disorders, metabolic syndrome, gut dysbiosis, bioactive dietary components

## Abstract

Gastrointestinal (GI) diseases, which include gastrointestinal reflux disease, gastric ulceration, inflammatory bowel disease, and other functional GI disorders, have become prevalent in a large part of the world population. Metabolic syndrome (MS) is cluster of disorders including obesity, hyperglycemia, hyperlipidemia, and hypertension, and is associated with high rate of morbidity and mortality. Gut dysbiosis is one of the contributing factors to the pathogenesis of both GI disorder and MS, and restoration of normal flora can provide a potential protective approach in both these conditions. Bioactive dietary components are known to play a significant role in the maintenance of health and wellness, as they have the potential to modify risk factors for a large number of serious disorders. Different classes of functional dietary components, such as dietary fibers, probiotics, prebiotics, polyunsaturated fatty acids, polyphenols, and spices, possess positive impacts on human health and can be useful as alternative treatments for GI disorders and metabolic dysregulation, as they can modify the risk factors associated with these pathologies. Their regular intake in sufficient amounts also aids in the restoration of normal intestinal flora, resulting in positive regulation of insulin signaling, metabolic pathways and immune responses, and reduction of low-grade chronic inflammation. This review is designed to focus on the health benefits of bioactive dietary components, with the aim of preventing the development or halting the progression of GI disorders and MS through an improvement of the most important risk factors including gut dysbiosis.

## 1. Introduction

Gastrointestinal (GI) disorders, whose prevalence has increased over the last few decades, are characterized by physiological and morphological abnormalities of the GI system that often occur in combination and include motility disorders, visceral hypersensitivity, altered mucosal and immune function, and altered intestinal microbiota [1,2]. Among GI disorders, inflammatory bowel diseases (IBD), including Crohn’s disease (CD), a chronic bowel disease that causes patches of inflammation in the GI tract [3], and ulcerative colitis (UC), which affects only the inner wall of the colon, are the most serious diseases [4]. Other common, idiopathic and chronic inflammatory disorders of the GI tract include diverticular disease, a chronic condition of small pockets of bowel, and irritable bowel syndrome (IBS) defined as an “abdominal discomfort associated with altered bowel habits” [5]. The main causes of GI disorders are genetic predisposition to the disease, pharmacological therapies (i.e., antibiotics, not limited to those administered for human use but also potentially including those used in farm animals and crops and ingested with the resultant foods), non-steroidal anti-inflammatory drugs (NSAIDs) (i.e., aspirin, ibuprofen, diclofenac), and unhealthy lifestyles, including irregular eating, physical inactivity, smoking, and low fiber diets [6,7,8,9]. Moreover, about 50% global population is affected by *Helicobacter pylori*, a Gram-negative bacterial pathogen, which might cause IBD and functional GI disorders [10]. All GI disorders commonly manifest abdominal pain, constipation, diarrhea, abdominal distention, gastric acidity, gastrointestinal reflux disease (GERD), GI tract (GIT) bleeding, malabsorption or malnutrition, and intestinal obstruction [11,12]. Some of the drugs currently in practice for the treatment of GI disorders include laxatives, anti-diarrheals, opioids, anti-emetics, motility enhancers, and anti-acidity, anti-ulcer, and anti-inflammatory agents [13]. Conventional therapies for IBD include corticosteroids, immunosuppressants and anti-tumor necrosis factor (TNF)-α antibodies, often correlated with a risk of opportunistic infections and dysplasias, with expensive consequences on health system management [14,15]. Evidence suggests that the Western pattern diet (WPD) has led to the wide spread of GERD. Studies have shown the prevalence of GERD to be 18.1–27.8% (North Americans), 8.8–25.9% (Europe), 11.6% (Australia), 23% (South America), 2.5–7.8% (East Asia), and 8.7–33.1% (Middle East) [16]. The prevalence of dyspepsia may vary from country to country, however, the pooled prevalence is reported by Ford et al. to be 21% [17]. In the adult population of the United States, diarrhea results in over 128,000 hospitalizations and 3000 deaths [18,19]. Similarly, available literature suggests that 12% of the worldwide population has had constipation [20]. Nyrop et al. (2007) have estimated the average annual direct health care cost of some GI disorders, per patient population visiting GI clinic, as $5049 (IBS), $6140 (diarrhea), $7522 (constipation), and $7646 (abdominal pain) [21]. Despite recent progression in knowledge of pathophysiological mechanisms involved in GI disorders, their etiopathogenesis has not yet been completely clarified and there is no marker that can lead to their definitive diagnosis. Some etiological factors have been identified, such as visceral hypersensitivity, infections, genetic and epigenetic factors, stress, and changes in the intestinal microbiota leading to dysbiosis [22,23]. 

**Figure 1:**
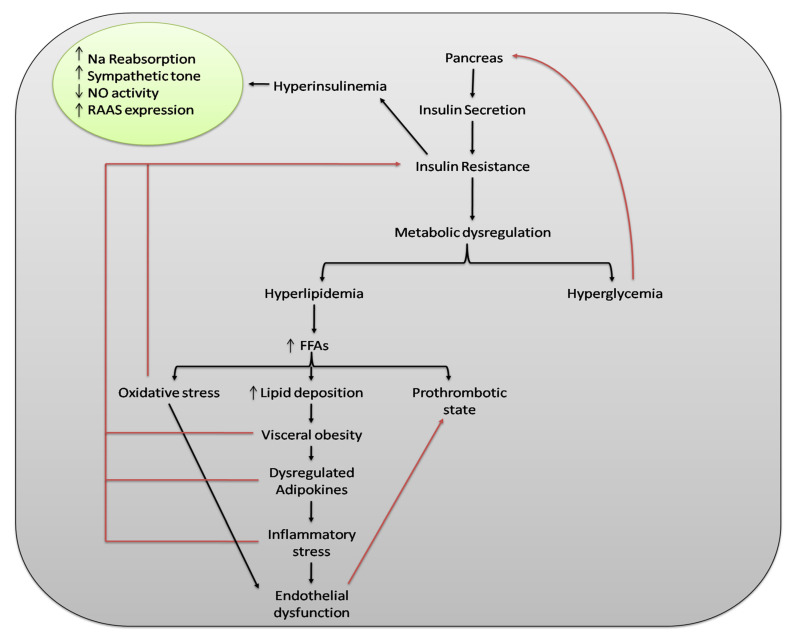
The impact of a dysregulated metabolism on the human body. The central element of metabolic syndrome (MS) is insulin resistance, which leads to metabolic dysregulation and eventually results in hyperglycemia and hyperlipidemia. Hyperglycemia stimulates β-cells of the pancreas and thus produces more insulin, which causes hyperinsulinemia. Hyperinsulinemia increases sympathetic tone, RAAS expression, and sodium reabsorption through nephrons while decreasing NO activity. Dysregulated fat metabolism results in increased production of FFAs and thus lipid deposition increases, resulting in visceral obesity. Visceral obesity causes dysregulation of adipocytokines and pro-inflammatory processes and leads to inflammatory stress. Increased production of FFAs also causes oxidative stress and prothrombotic states via other mechanisms. Oxidative and inflammatory stresses may lead to endothelial dysfunction, which may further contribute to a prothrombotic state. Oxidative stress, visceral obesity, dysregulated adipocytokines, and pro-inflammatory cytokines further contribute to insulin resistance. Increase (↑); decrease (↓); sodium (Na); nitric oxide (NO); renin-angiotensin-aldosterone system (RAAS); free fatty acids (FFAs).

Metabolic syndrome (MS) is a condition of low-grade chronic inflammation due to both genetic and environmental factors, including a number of risk factors for serious diseases such as hyperglycemia, abdominal obesity, hyperlipidemia, and hypertension [24,25]. The clinical significance of MS was highlighted by Reaven in 1988, while describing the role of insulin resistance in human disease [26]. An untreated and persistent state of MS may lead to a number of pathologies, the most common of which are cardiovascular disorders (coronary heart disease, cardiac failure, and stroke), type 2 diabetes mellitus, and hepatic abnormalities such as hepatic steatosis [27,28,29]. As reported by Sadeghi, a variety of metabolic disturbances, including hypertension, hyperglycemia, and gout, were already described in the 1920s [30]. Later, in 1947, Vague described the obesity phenotype as being affected by metabolic abnormalities, type 2 diabetes mellitus (T2DM), and cardiovascular disease [31]. The WHO definition and the European Group for the Study of Insulin Resistance agree over the inclusion of glucose intolerance and insulin resistance as essential components of MS [32,33]. Several definitions of said syndrome are in use, but since 2004, the International Diabetes Federation (IDF) has established a unified definition of MS, highlighting the key role of obesity as a risk factor for the diseases reported above [34]. The main risk factors for the development of MS are positive family history [35], smoking [36], excessive alcohol intake [37], aging, low social-economic status, postmenopausal status [38], sedentary life style [39], unhealthy dietary patterns [40], and intake of some medications (atypical antipsychotics) [41]. Figure 1 shows the impact of a dysregulated metabolism on the human body by illustrating the pathogenic pathway of MS. Metabolic disorders share common pathophysiological mechanisms and thus different pharmacologic entities are used in combination for therapeutic purposes [42]. Effective interventions may include physical exercise, dietary modifications, and pharmacological agents such as insulin sensitizers, renin-angiotensin-aldosterone system (RAAS) inhibitors, statins, and fibrates [43,44]. Over the past two decades, a large worldwide increase in people with MS has taken place, associated with a global epidemic of obesity and T2DM. About 20–25% of the world adult population is living with MS [45]. WHO estimates that about 650 million people have been living with obesity, 422 million with diabetes, and 1.13 billion with hypertension [46,47,48].

**Figure 2:**
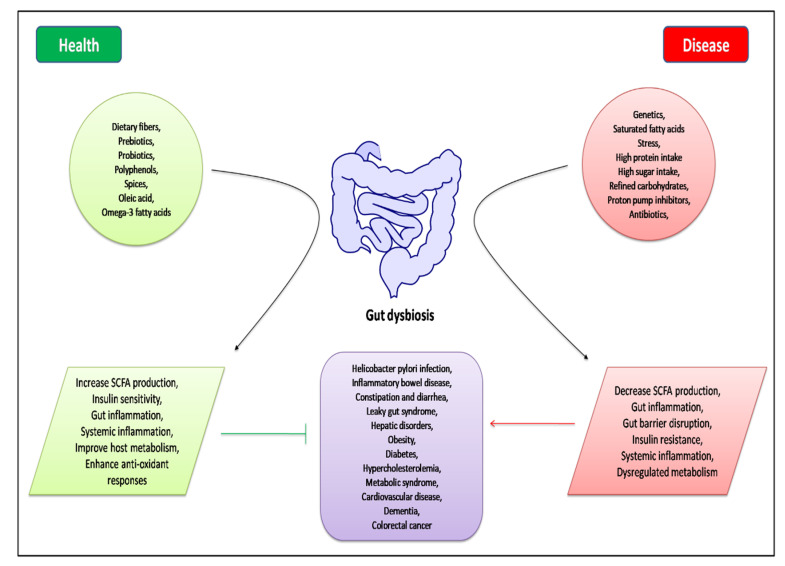
Schematic representation of intrinsic and extrinsic factors responsible for gut dysbiosis, and their role in health and disease.

Growing evidence suggests that dysbiosis of the gut microbiota, due to multiple intrinsic and extrinsic factors such as genetic variations, diet, stress, and pharmacological therapy [49], is associated with the pathogenesis of both intestinal and extra-intestinal diseases (Figure 2). In fact, the interaction between microbiota and the host immune system is considered to be a key element for a new understanding of the pathogenesis of a large spectrum of diseases including GI disorders and MS [50]. Current studies highlight the role of a healthy gut microbiota in modulating the onset of various GI diseases such as IBD, colon cancer, celiac disease, and IBS. In addition, dysbiosis is one of the driving factors of a dysregulated metabolism. Hur et al. report the important role of gut microbiota in the pathogenesis of T2DM by influencing body weight, pro-inflammatory activity, and insulin resistance [51]. A pivotal role of gut microbiota is the fermentation of dietary polysaccharides that the human body cannot otherwise digest. Dietary fibers consist of the indigestible portion of plant food carbohydrates, containing insoluble and soluble fibers. Soluble fibers are digested by enzymes derived from the gut microbiota into short-chain fatty acids (SCFAs). SCFAs (butyrate, acetate, and propionate) are absorbed in the intestines and used as energy by the host. In addition to their role as energy substrates, SCFAs act as regulators of food or energy intake and inflammation [52,53]. In particular, butyrate promotes regeneration and protection of intestinal cells, the production of mucin, the reduction of hypercholesterolemia levels, as well as the release of hormones and/or neurotransmitters important for the regulation of intestinal motility and of insulin resistance [54]. An improvement in abdominal pain following the intake of butyrate has been observed in IBS patients due to an alteration in neurotransmitter release and a reduction in the hypersensitivity of intestinal mechanoreceptors, which may result in decreasing luminal pressure and/or peristalsis [55,56].

Today, lifestyle and diet are recognized as the cornerstones of prevention of pathologies such as cardiovascular diseases, T2DM, MS, and GI disorders. Moreover, the modulation of intestinal dysbiosis through dietary supplements, according to the latest evidence, is used to restore the equilibrium of gut microbiota. Considering that dysbiosis can be a common link between GI disorders and MS, and a correct diet can be used to restore the altered microbiota and is considered to be the first approach to treat both GI disorders and MS, the aim of this review is to summarize the current knowledge of the protective roles of functional dietary components in GI and MS, and to assist in the derivation of a general perspective of these broad areas.

## 2. Methodology

**Figure 3:**
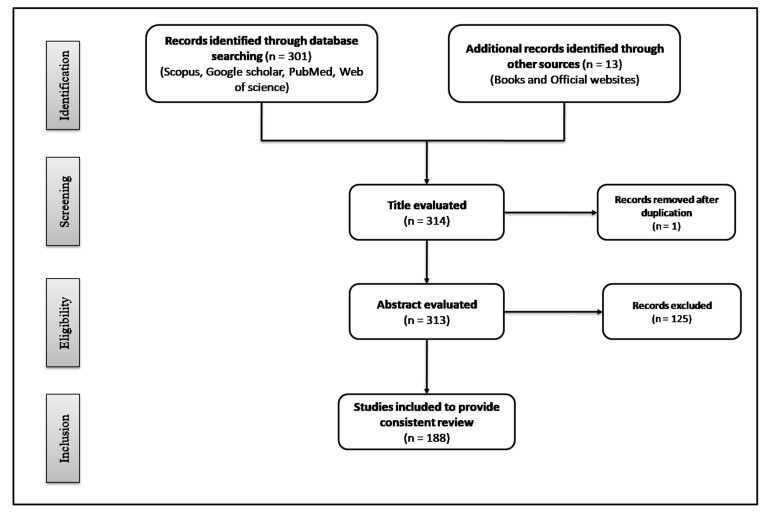
PRISMA flow diagram, showing the process of study selection.

The present study consists of an up-to-date review of the literature covering the health benefits of functional dietary components with special reference to gut microbial modulation in GI disorders and MS. Various electronic databases were used for the literature search, including Scopus, Google scholar, PubMed and Web of Science, using the keywords “metabolic syndrome”, “gastrointestinal disorders”, “dietary food components”, “nutraceuticals”, “functional foods”, “dietary fibers”, “probiotics”, “prebiotics”, “saturated fatty acids”, “short chain fatty acids”, “monounsaturated fatty acids”, “polyunsaturated fatty acids” “polyphenols”, and “spices”. The criteria for selecting articles were “studies reported in English because of language barriers” and, “pre-clinical and clinical studies related to dietary food components”. The results returned 301 papers published up to the 2020 date. Of these articles, 175 were selected, summarized, and critically discussed so as to provide a consistent review. Some books and official websites (World Health Organization and, Food and Drug Organization) were also used for citing specific data within the scope of present study. Figure 3 illustrates the PRISMA flow diagram for study selection. In the following sections, functional dietary components are discussed, with their positive role in GI disorders and MS through the modulation of their most important risk factors, including gut microbes.

## 3. Bioactive Dietary Components and Gastrointestinal Disorders

The health of the digestive system is important for its appropriate physiological functions, where said physiological parameters largely depend on the type of food ingested and the presence of bioactive components therein [57]. The major factors associated with the development of GI disorders are the use of medications for the treatment of chronic disorders [58], cultural attitudes [59], and socioeconomic factors [60]. The undesirable effects of drugs used for the treatment of GI disorders have led scientists and clinicians to focus on the use of alternative options. The use of dietary approaches, functional foods, and food supplement-based approaches are core parts of alternative treatments [61]. The potential roles of functional dietary components in GI disorders are summarized in Table 1.

### 3.1. Dietary Fibers

Dietary fiber is the portion of plant-derived food that cannot be completely broken down by human digestive enzymes, but can be digested by microflora in the gut. Dietary fiber includes non-starch polysaccharides (NSP) such as celluloses, hemicelluloses, gums and pectins, resistant dextrins, and resistant starches. In addition to NSP, dietary fiber includes lignin, which is a complex non-carbohydrate aromatic cross-linked polymer. Dietary fiber can be categorized into two main classes: insoluble dietary fiber (celluloses, some hemicelluloses, and lignins) and soluble dietary fiber (β-glucans, pectins, gums, mucilages, and hemicelluloses). Foods rich in dietary fiber include whole grains, some fruits and vegetables, oats, barley, and beans [81,82]. Soluble dietary fibers are fermented in the colon to a greater extent to produce by-products such as short chain fatty acids (SCFAs) and possess water holding capacity. In contrast, insoluble fibers are subjected to limited fermentation with minimal water holding capacity. In addition, insoluble fibers increase fecal mass and accelerate colonic transit time [83,84,85]. 

Daily consumption of dietary fiber in sufficient amounts may confer benefits to human health. Soluble fibers may accelerate oro-anal transit by reducing intra-colonic pressure and thus may improve functional GI disorders. These effects may be secondary to the effects of SCFAs on the modulation of microbiota such as enrichment of Firmicutes, Actinobacteria, and Bacteroidetes, and alteration of low-grade inflammation [62,86,87,88]. The acetate, propionate, and butyrate byproducts of fermentation can decrease colonic pH, which favors the growth of beneficial microbes [63]. Butyric acid has an important role in gastrointestinal function. Some in vivo and in vitro studies suggest that butyric acid may be useful in the treatment of ulcerative rectocolitis. In fact, low concentrations of short chain fatty acids, acetyl CoA, and pantothenic acid have been found in patients with ulcerative rectocolitis [89]. Butyrate could act as an epigenetic “switch” capable of stimulating the immune system through the induction of the production of regulatory T cells in the intestine. Moreover, it has been reported to suppress interferon-γ (IFN-γ)-mediated inflammation [64]. This finding provides evidence to support the possible use of butyrate as a therapy for IBD, such as Crohn’s disease [65]. Moreover, butyrate increases passive absorption of water in the colon and may prevent diarrhea [66].

#### Prebiotics

Many dietary fibers are classified as prebiotics, with a beneficial role in GI disorders [90]. These are compounds that induce the growth or activity of beneficial microbes contributing to the physical well-being of their host [91,92]. Some common examples of prebiotics include lactulose, inulin, psyllium, and other oligosaccharides (fructo-oligosaccharide or FOS and galacto-oligosaccharide or GOS). Asparagus, garlic, onions, artichoke, leeks, tomatoes, bananas, oats, soy beans, and wheat are dietary sources rich in prebiotic contents [93,94]. The regular intake of prebiotics may stimulate the intestinal immune system, control the growth of pathogens and toxins, enhance the production of SCFAs, reduce lactose intolerance, and decrease constipation [67]. Some GI disorders such as IBS and Crohn’s disease are associated with an increase in mucosal inflammation and a decrease in several gut bacteria, and low dose prebiotic supplementation has been reported to reverse the pathogenesis of both disorders by downregulating the mucosal inflammatory cascade and increasing eubiotic gut bacteria. However, larger doses may have neutral or negative effects [68]. The term synbiotics is used for the formulation of nutritional supplements containing combinations of probiotics and prebiotics designed for their synergistic health benefits [69].

### 3.2. Probiotics

Probiotics can be defined as live microorganisms consumed as food supplements or in functional foods, providing health benefits when consumed in adequate amounts generally by improving or restoring the gut flora [95]. Probiotics usually include species of *Lactobacillus*, *Bifidobacterium*, or *Saccharomyces* genera, some Gram-positive cocci and some strains of *Escherichia coli*. They can be consumed in powdered, gel, paste, or granule forms, in liquid form and in capsule forms, have non-pathogenic and non-toxic properties, and are able to adhere to gut epithelial tissues and produce SCFAs [96,97,98]. Probiotics can provide a number of benefits to the host beyond nutrition, such as enhancing intestinal epithelial integrity, regulating immunity of the GI mucosa, protecting gut barrier disruption, and inhibiting the growth and/or activity of pathogenic microbes in the GI [70,71,99]. Commercially available probiotic formulations should contain the optimal number of colony forming units (CFU) for each bacterial strain above the critical threshold (10^6^ CFU), with some available formulations containing up to 10^12^ CFU. Although the optimal number of CFU for each bacterial strain delivered remains unknown, daily probiotic doses of about 10^6^–10^9^ are recommended for their beneficial effects [100,101].

Due to their multiple benefits, selected strains of probiotics can be used to treat some common conditions associated with the GI tract [102,103,104,105]. Waller et al. (2011) demonstrated the impact of *Bifidobacterium lactis* HN019 on whole gut transit time and functional GI symptoms in an adult population [106]. A total of 100 adults (mean age: 44 years) with functional GI symptoms were recruited for the study and were allowed to consume *B. lactis* HN019 strain in a high dose of 17.2 billon CFU, a low dose of 1.8 billion CFU or a placebo for 14 days. The results were significant at both high and low doses, and showed that supplementation with *B. lactis* HN019 strain results in decreased whole gut transit time and improvement of functional GI symptoms, with no adverse events reported. Srinarong et al. (2014) observed an improvement in the eradication rate of *H. pylori* by standard triple therapy through addition of bismuth and a probiotic supplement [72]. The study was performed in Thailand where clarithromycin resistance is a potential problem in the treatment of *H. pylori* infections with standard therapy. *H. pylori* infected patients (100 subjects) were randomized and received 7 or 14 days standard triple therapy (lansoprazole 30 mg twice daily, amoxicillin 1 g twice daily, clarithromycin MR 1 g once daily) plus bismuth subsalicylate (1.048 mg twice daily) and probiotic supplements (composed of *B. lactis*, *Lactobacillus acidophilus*, and *L. paracasei*) or placebo. The eradication rate of *H. pylori* was 100% in patients treated with 7 or 14 day probiotic supplementation. 

Almeida et al. (2012) supplemented 27 lactose intolerant patients with a probiotic product containing *Lactobacillus casei* Shirota and *Bifidobacterium breve* Yakult (10^7^–10^9^ CFU of each strain) to study the beneficial effects of probiotic supplementation in lactose intolerance [73]. Twenty-seven subjects were recruited in the study and were supplemented with probiotic product for 4 weeks. It was noted that probiotic supplementation improved the symptoms of lactose intolerance and decreased hydrogen production (produced when undigested lactose ferments in colon) as reflected by breath hydrogen concentration. Xue et al. (2017) determined the in vivo effects of probiotic supplementation on the progression of non-alcoholic fatty liver disease (NAFLD) [74]. Eight-week-old male Sprague Dawley rats were treated for 12 weeks with standard diet, high-sucrose high-fat (HSHF) diet supplemented with probiotics (0.5 g/day/rat). Probiotic supplementation consisted of 0.5 × 10^6^ CFU of *Bifidobacterium infantis* and *Lactobacillus acidopilus*, and 0.5 × 10^5^ CFU of *Bacillus cereus*. Blood and tissue samples (liver and intestine) were collected at the end of the treatment period for biochemical and histological examination. The results showed that probiotics improved dysbiosis of intestinal flora, reduced body weight, ameliorated loss of intestinal barrier integrity, reduced circulating inflammatory cytokines and ameliorated hepatic pathology (hepatocyte swelling and inflammatory cells infiltration).

### 3.3. Polyphenols 

Polyphenols represent a group of bioactive phytochemicals distributed throughout the higher plants and found in a variety of fruits, vegetables, seeds, and beverages and in minor quantities in dry legumes and cereals [107]. They are secondary metabolites and, with over 10,000 structural variants, comprise a wide variety of molecules with a polyphenolic structure. They are divided into flavonoids (flavonols, flavanones, flavanols, flavones, isoflavones, and anthocyanidins) and non-flavonoids (phenolic acids, stilbenes, coumarins, xanthones, lignans, and curcuminoids) [108,109,110,111]. They are the most abundantly consumed antioxidants, as Western populations may take polyphenols in quantities of up to 1 g/day [112]. In recent decades they represent a topic of great scientific attention due to their potential human health benefits [113]. 

Polyphenols are known to possess antioxidant and anti-inflammatory effects and thus can be considered as alternative protective agents against chronic inflammatory conditions of the GI tract [114]. There are two main reciprocal interactions of polyphenols with the gut microbiome that consist of modulation of gut microbiota by dietary polyphenols, and production of bioactive metabolites of polyphenols by gut microbial species [76]. As it is currently understood, polyphenols suffer from low bioavailability, and gut microbes may help polyphenols in their absorption from the GIT and thus may increase their bioavailability [115,116]. Larrosa et al. (2009) investigated the effects of dietary resveratrol on colon microbiota, inflammation, and tissue damage in dextran sulfate sodium (DSS)-induced experimental colitis in rats [75]. Rats were supplemented with 1 mg/kg/day dose of resveratrol for 25 days, where 5% DSS was used to induce colitis in the last 5 days. It was noted that resveratrol diminished the growth of enterobacteria while increasing *Lactobacilli* and *Bifidobacteria*. It significantly reduced the inflammatory markers, prostaglandin E2, COX-2, prostaglandin E synthase, and nitric oxide levels in colon.

Evidence also supports the anti-*H. pylori* effects of polyphenols such as EGCG, as examined by Lee et al. (2004) using cultured gastric cells contaminated with *H. pylori* bacteria. It was concluded that pretreatment with a low dose EGCG significantly attenuated bacterial induced cytotoxicity, altered the mitogen activated protein kinase (MAPK) signaling pathway and reduced apoptosis. However, it was noted that higher dose EGCG (but lower than 250 µmol/L, which showed significant cytotoxic effects against gastric cancer cells) resulted in increasing apoptosis [77]. 

The host microbiota plays a crucial role in producing polyphenol components with increased bioactivity, and the importance of the gut microbiome in polyphenol metabolism has been highlighted by the fact that no such metabolites form for catechin, apigenin, myricetin, hesperidin, rutin, and naringinin germ-free or antibiotically treated animals [117,118]. Urolithin produced from ellagic acid by *Gordonibacter urolithin faciens* and *Gordonibacter pamelaeae*, possesses anti-inflammatory and anti-oxidant properties, and can influence intestinal inflammation [119,120]. The production of nutraceuticals and functional foods with the addition of urolithin producing probiotic strains is highly suggested, with the aim of improving the health benefits of ellagitannins [121]. The *Humulus lupulus* L. (hops) is a primary agent in the production of beer, containing phenyl flavonoids most predominantly isoxanthohumol, with weak anti-estrogenic activity and anti-carcinogenic effects as tested using in vitro models of colon cancer [122]. The gut microbiota coverts isoxanthohumol into more strong estrogenic compound 8-prenylnaringenin; however, the difference in the anti-carcinogenic effects of both compounds have been reported not to be significant [123].

### 3.4. Spices 

Spices are widely used in traditional medicine, in foods as preservatives, as natural colors, in cosmetics as perfumes, and in dental preparations, thus playing a significant role in the economy of the countries of production. Recent research reveals that dietary spices in their minute quantities have a great influence on human health due to their antioxidant, chemopreventive, anti-mutagenic, anti-inflammatory, and immune modulatory effects [124,125,126]. Several spices have been used in traditional and Indian systems of medicine against a number of digestive disorders. They have been shown to stimulate digestive action, most probably by enhancing the activity of digestive enzymes and stimulating bile secretion from liver [127]. However, the evidence from available literature of the beneficial effects of spices in GI health via gut microbial modulation are still very limited; and only few studies have shown the link between spices and the alteration of intestinal flora in GI disorders.

Curcumin oral consumption may exert regulatory actions on gut microbiota including intestinal microbial richness, diversity, and composition, which could be responsible for its pharmacological effects [128]. Nanoparticle formulations have been developed to overcome the poor bioavailability of curcumin, which could increase intestinal butyrate producing bacteria to a greater extent and could improve gut mucosal permeability [129]. Ohno et al. (2020) demonstrated the effects of nanoparticle curcumin on modulation of gut microbiota and induction of regulatory T cells in ameliorating DSS induced colitis [78]. It was noted that curcumin nanoparticles significantly decreased body weight, disease activity index, and histological colitis score, improving mucosal permeability and suppressing NF-κB activation. Treatment with curcumin nanoparticles markedly increased the abundance of butyrate producing bacteria and fecal butyrate concentration. The production of *Clostridium cluster* IV, *Clostridium cluster* XI, and *Clostridium subcluster* XIVa was significantly increased while the production of *Lactobacillus* significantly decreased in the curcumin nanoparticle group, compared to the control group. Moreover, increased expansion of CD4+ Foxp3+ regulatory T cells and CD103+ CD8α− regulatory dendritic cells in the colonic mucosa was observed in the curcumin nanoparticle treated group.

Botschuijver et al. (2018) reported in their in vivo experimental study that supplementation of Menthacarin^®^ (a combination of essential oils from peppermint and caraway) mediated reversal of visceral hypersensitivity and pain in IBS, may be associated with a modulation of the microbiome and mycobiome [79].The key results of the study included attenuation of visceral hypersensitivity and pain, and inhibition of microbial species (*Candida albicans* and *Bacillus subtilis*). Similarly Li et al. (2020) suggested that cinnamon essential oil can be used as a preventive or therapeutic agent in IBS, while exploring its effects on the gut microbiota in a mouse model of DSS induced colitis [80]. Briefly, the results of the study indicated that treatment with cinnamon essential oil effectively alleviated DSS induced colitis, with improved diversity and richness of intestinal microbiota. A decrease in *Bacteroides* and *Helicobacter* spp. and an increase in SCFAs producing bacteria (*Alloprevotella* and *Lachnospiraceae_NK4A136*-group) was observed in mice treated with cinnamon essential oil. Moreover TNF-α and toll-like receptor (TLR4) expression was correlated with intestinal microbial species, and it was concluded that both of these factors were inversely correlated with SCFAs producing bacteria.

## 4. Bioactive Dietary Components and Metabolic Syndrome

Changes in nutritional composition of the diet and in energy expenditure can lead to an unhealthy state characterized by a low intake of dietary fibers and polyunsaturated fatty acids and a high consumption of sugars, total fats, cholesterol, and refined carbohydrates, creating an alarming situation of increasing incidence of MS risk factors. Traditional dietary approaches are known to play a substantial role in improving the overall health of an individual and in the prevention of metabolic dysregulation, but these may lack efficacy in achieving long term goals, possibly due to poor compliance. Thus, science has turned its attention towards nutrients with the ability to modulate gut microbiota or other biochemical pathways, with the ultimate goal of preventing MS. These include dietary fibers, prebiotics, probiotics, polyunsaturated fatty acids, and polyphenols [130,131,132]. Table 2 summarizes the benefits of functional dietary components in MS.

### 4.1. High Fiber Diet

There is a general observation that the obese and diabetic population of North America lacks an adequate amount of fiber in their daily diet. Studies have also shown that a daily intake of dietary fibers, fruits, and legumes possesses an inverse relationship with metabolic dysregulation and related consequences [157,158]. There is no special recommendation for a daily intake of dietary fibers by individuals at high risk of diabetes and other metabolic diseases. The American Diabetes Association (ADA) recommends the daily intake of dietary fibers as 14 g fibers per 1000 kcal daily or 38 g/day for men and 25 g/day for women [159]. Of particular interest, dietary fibers could play a role in the prevention and/or treatment of metabolic dysregulation through different mechanisms related to their specific physical properties, chemical structure, or fermentability in the gut by intestinal flora. A regular intake of dietary fibers in sufficient amounts can improve insulin sensitivity, metabolic homeostasis, and endothelial dysfunction, prevent against obesity, and regulate inflammatory biomarkers [160,161].

Kjølbæk et al. (2020) investigated arabinoxylan oligosaccharides and polyunsaturated fatty acids for their effects on gut microbiota and metabolic markers [133]. Twenty seven overweight individuals with MS signs participated in a 12 week randomized cross-over trial, divided into two treatment periods of 4 weeks each with a washout period also of 4 weeks. The resulting analysis of arabinoxylan oligosaccharide intervention revealed an increased abundance of species of the phyla Bacteroidetes and Firmicutes, including *Bifidobacteria* species (*Bifidobacteria faecale*, *B. stercoris*, *B. dolescentis*), *Eubacterium rectale*, *Eubacterium hallii*, *Faecalibacterium prautsnitzii*, *Dorea longicatena*, *Blautia luti,* and *Blautia wexlerae* while the relative abundance of *Clostridium methylpentosum*, *Anaerotruncus colihominis,* and *Erysipelothrix rhusiopathiae* decreased. No effects of PUFAs were observed on gut microbiota. Moreover, the gut microbial modulation by arabinoxylan oligosaccharides was positively correlated with blood concentration of insulin, markers of lipid metabolism (triglycerides, low density lipoprotein, very low density lipoprotein, total cholesterol, and apolipoprotein B), hepatic function (Alanine aminotransferase) as well as HOMA scale of insulin resistance (HOMA-IR) and HOMA scale of beta cell function (HOMA-β).

Li et al. (2019) explored the effects of inulin to alleviate T2DM via anti-inflammation and gut microbial modulation in db/db mice [134]. The mice were divided into different groups and were supplemented with a standardized diet containing 5% inulin, or dietary fiber powder containing 91% inulin, and a 9% mixture of glucose, sucrose, and fructose, for 6 weeks. Serum analysis of inulin treated groups showed a significant decrease in body weight, fasting blood glucose, glycated hemoglobin, plasma lipopolysaccharide, and alteration of inflammatory mediators such as decreased IL-6, TNF-α, and IL-17A, increased IL-10. Statistical analysis of gut microbiota using stool culture revealed elevated *Bacteroides* and *Cyanobacteria*, with a reduction of *Ruminiclostridium_6*, *Deferribacteres*, *Mucispirillum*, and *Tenericutes* in inulin treated groups. It was noted upon correlation analysis that *Bacteroides* and *Cyanobacteria* positively correlated with anti-inflammatory IL-10 while *Deferribacteres*, *Tenericutes*, *Mucispirillum*, and *Ruminiclostridium_6* were closely related to inflammatory mediators TNF-α, IL-6, and IL-17A. Furthermore *Ruminiclostridium_6* and *Mucispirillum* were also positively correlated with plasma lipopolysaccharide. The authors concluded that dietary inulin can halt the progression of diabetes via suppression of the inflammatory cascade through gut microbial modulation.

#### Prebiotics

As mentioned before, many dietary fibers are classified as prebiotics, and these generally possess indirect health benefits stimulating the growth of *Lactobacillus* and *Bifidobacterium* species in the gut, thus improving metabolism of the host [135]. Oligofructose administration to mice with altered gut microbiota and induced metabolic dysregulation resulted in an increase in glucose tolerance, decrease in insulin resistance, and upregulation of the expression of anti-inflammatory cytokines [136]. Animal studies suggest that supplementation with prebiotics can positively regulate body weight and insulin sensitivity. Through alteration of genetic expression by acting on peroxisome proliferator-activated receptor gamma (PPAR-γ) and GPR43, prebiotics can enhance the metabolic response to leptin, increase lipolysis and decrease adipogenesis [137,162,163]. In a randomized controlled trial on 55 female subjects (30 subjects in intervention group and 25 in control group) with T2DM, Farhangiet al. showed that resistant dextrin (10 g/day for 8 weeks) exerts beneficial effects on immune-mediated inflammation and the hypothalamic–pituitary–adrenal axis in treated women in comparison with control group women. In particular resistant dextrin induces a significant decrease in levels of cortisol, KYN, KYN/TRP ratio, IFNγ, IL12, IFNγ/IL10 ratio, LPS, and a significant increase in the monocyte, GHQ, DASS, CD8, IL10, IL4. Moreover, in the same study the influence of prebiotic supplementation on depression, anxiety, and stress was evaluated. The registered mood and metal health improvement was ascribed to the changes induced in the gut microbiota profile, which was depleted of pathogenic bacteria such as *Clostridia* species and enriched with microorganisms associated with the regulation of gamma-aminobuytic acid (GABA) receptors through the vagus nervous system, and the release of cortisol, such as *Bifidobacterium* and *Faecalibacterium* species, exerting anti-inflammatory activity blocking NF-kβ activation [164].

### 4.2. Probiotics

Gut dysbiosis possesses potential effects on metabolic health ranging from insulin resistance and glucose intolerance to MS. Disturbances in intestinal microbiota could lead to β cell dysfunction, shifts in energy metabolism, fat synthesis, development of adipose tissues, and systemic inflammation [165,166]. Gut microorganisms are known to play a role in energy absorption, storage, and consumption following dietary intake. Modulation of gut microbiota could be a potential target to prevent or reverse dysmetabolic states in individuals, including obesity, T2DM, MS, and NAFLD [167,168,169]. Sroka-Oleksiak et al. (2020) performed a metagenomic analysis of intestinal microbiota in obese and T2DM patients. Obese patients qualifying for bariatric surgery were included in the study [138]. The results of analysis showed no significant change in the phyla Firmicutes, Proteobacteria, and Actinobacteria among diseased individuals when compared to healthy ones. However, considerable changes were observed on the microbiological core of the genus *Bifidobacterium*, as significantly lowered levels of *Bifidobacterium* species were observed in patients with obesity and obesity with T2DM. The authors suggested considering the genus *Bifidobacterium* as a potential biomarker in the progression of obesity and T2DM. Liu et al. (2020) observed the protective effects of probiotics against MS through modulation of hepatic peroxisome proliferator activated receptor alpha–fibroblast growth factor 21 (PPARα–FGF21) signaling in a mouse model of high-fat high-fructose (HFHF) diet [139]. The addition of a *Lactobacillus rhamnosus* GG culture supernatant to the HFHF diet resulted in an improvement in body weight, insulin resistance, glucose intolerance, and hepatic steatosis. Upregulation of PPARα–FGF21 signaling and increases in fecal butyrate concentration were suggested to be responsible mechanisms for improvement of MS parameters. 

Wang et al. (2020) studied the potential of composite probiotics to alleviate T2DM by modulating intestinal microbiota and enhancing the expression of glucagon like peptide-1 (GLP-1) in db/db mice [140]. The research team isolated 14 probiotic species from fermented camel milk including 10 *Lactobacillus* strains (*Lactobacillus helveticus*, *Lactobacillus plantarum*, *Lactococcus lactis*, *Lactobacillus paracasei*, *Lactobacillus pentosus*, *Lactobacillus paracasei* subsp. *tolerans*, *Lactobacillus hilgardii*, *Lactobacillus rhamnosus*, *Lactobacillus harbinensis*, and *Lactobacillus mucosae*) and four yeast species (*Candida ethanolica*, *Issatchenkia orientalis*, *Pichia membranifaciens,* and *Kluyveromyces marxianus*). The high dose of probiotics contained 1 × 10^10^ CFU/mL of *Lactobacillus* strains and 1 × 10^8^ CFU/mL of yeast strains, while the low dose probiotics contained 1 × 10^8^ CFU/mL of *Lactobacillus* strains and 1 × 10^6^ CFU/mL of yeast strains. Treatment with probiotics at both high and low doses resulted in increases in SCFA concentration, improvement of intestinal barrier function, protection of pancreas against apoptosis, and positive regulation of metabolic parameters, including upregulation of G protein-coupled receptor 43/41 (GPR43/41), proconvertase 1/3, and proglucagon activity, resulting in enhanced glucose-triggered GLP-1 and insulin secretion. The protection of the pancreas against apoptosis by treatment with probiotics might be related to the upstream regulation of the PI3K/AKT pathway.

Michael et al. (2020) conducted randomized, double blind, placebo controlled studies to observe the anti-obesity effects of probiotic supplementation (*Lactobacilli* and *Bifidobacteria*) in overweight and obese adults [141]. A total of 220 subjects were recruited for the study, where the inclusion criteria for subject selection was defined as age between 30 and 65 years, waist circumference >100 (men) or >89 (women), body mass index (BMI) between 25 and 34.9 kg/m^2^, and subjects having received no statin therapy in the last 3 months. The probiotic formula per capsule was comprised of *Lactobacillus acidophilus* CUL21, *Lactobacillus acidophilus* CUL60, *Lactobacillus plantarum* CUL66, *Bifidobacterium animalis* subsp. *Lactis* CUL34, and *Bifidobacterium bifidum* CUL20. Patients were asked to take one capsule daily for 180 days at any time of the day, with or without food, and not to take them within 2 h of any antibiotic, if any were taken. Significant reduction in body weight, BMI, waist circumference, and waist to hip ratio, and a greater decrease in LDL cholesterol was observed. Additionally, changes to physical and biochemical parameters recorded at 3 months were not considerable, suggesting that a 6 month dosing of the abovementioned probiotic formula is essential to achieve clinical benefits.

### 4.3. Fatty Acids

A high-fat diet and circulating free fatty acids (FFAs) are known risk factors for causing insulin resistance, visceral obesity, and MS. In the past decade, several studies have explored the role of specific dietary fats in gut dysbiosis with relation to MS risk factors [170]. Saturated fatty acids promote insulin resistance via numerous mechanisms including stimulation of Toll-like receptor (TLR) and Jun N-terminal kinase (JNK) activity [171,172]. However, the direct effects of saturated fatty acids are less pronounced than the influence of the gut microbiome on the host metabolism. Germ-free animals are protected against insulin resistance and obesity induced by a high-fat diet, as demonstrated by Bäckhed et al. (2004) and Ding et al. (2010) [173,174]. As evident from in vivo studies, saturated fatty acids have been shown to cause metabolic endotoxemia by causing overgrowth of *Bilophila wadsworthia* (bile-tolerant Gram-negative bacteria), *Enterobacteriaceae*, and *Escherichia coli*, with a substantial decrease in *Bifidobacteria* [175,176,177].

SCFAs (acetate, propionate, and butyrate) are saturated fatty acids with less than six carbon atoms, and possess positive effects on gut dysbiosis and the host metabolism [142]. SCFAs tend to reduce inflammation though activation of G-protein-coupled receptor 43 (GPR 43), which may lead to increased insulin sensitivity in liver and muscles, and increased energy expenditure [143]. SCFAs mainly originate from indigestible carbohydrates or prebiotics by microbial fermentation, and it is well documented that supplementation with prebiotics enhances butyrate production in Wistar rats and increases Bacteroidetes. On other hand, a high-fat diet reduces butyrate formation with increased liver fat and inflammation [178]. Prebiotics have additional benefits on MS risk factors by increasing the abundance of mucin-foraging bacteria (*Akkermansia muciniphila*) that were depleted in obese and diabetic mice [144]. Butyrate has anti-obesity effects through upstream regulation of the expression of angiopoietin-like protein-4 (ANGPTL4) in human epithelial cells, resulting in the reduced expression of lipoprotein lipase (LPL) and increased lipolysis [145].

Mujico et al. (2013) determined the effects of fatty acids on gut microbiota modulation in diet induced obese mice and it was noted that monounsaturated fatty acid (oleic acid) prevented high-fat diet induced dysbiosis and improved the abundance of *Bifidobacteria* [146]. In another study, supplementation of diet enriched in oleic acid in young adults resulted in improved insulin sensitivity [147]. Omega-6 fatty acids increased the abundance of bacterial groups associated with inflammation including *Enterobacteriaceae*, *Proteobacteria*, and segmented filamentous bacteria, along with weight gain and fatty infiltration of liver when supplemented to C57BL/6 mice [179,180,181]. After experimental evaluation of the effects of dietary fats on gut microbiota architecture and host inflammatory mediators in mice adipose tissues, Huang et al. reported that dietary omega-6 fatty acids can cause an increase in *Proteobacteria* with greater macrophage infiltration of adipose tissues and increase adipose expression. Furthermore the effects of omega-6 fatty acids observed by Huang et al. were more pronounced than those of saturated fatty acids [182].

In contrast, omega-3 fatty acids modulate gut microbiota, which in addition to other mechanisms may be responsible for their greater potential to prevent against metabolic dysregulations [183]. Ghosh et al. (2013) documented that gut dysbiosis caused by omega-6 fatty acids in aged mice was prevented by supplementation of omega-3 fatty acids (eicosapentaenoic acid and docosahexaenoic acid). Omega-3 fatty acids reduced bacterial overgrowth and recruited regulatory T cells to the small intestine, thereby reducing diet-induced inflammation [148]. A recent study has shown a decreased abundance of intestinal microbes associated with ulceration (*Helicobacter*), infection (*Pseudomonas*), and weight gain (Firmicutes) with fish oil supplementation in mice [149]. In addition to its positive effects on metabolic endotoxemia, omega-3 supplementation also increases insulin sensitivity by upregulating cell surface expression of GLUT4 and boosts insulin secretion from pancreatic β cells by activating G-protein-coupled receptor 40 (GPR 40) [150,151].

### 4.4. Polyphenols

Several polyphenol enriched foods and beverages, including green tea, berries, red wine, nuts, grape seeds, and dark chocolate, have been found to have significant effects on different aspects of metabolic dysfunction [184]. Flavanones, flavonols, isoflavone, anthocyanins, proanthocyanidins, and resveratrol are the most studied classes of polyphenols that possess beneficial health effects on MS risk factors, most probably through improvement of insulin signaling, downregulation of oxidative stress, gut bacteria modulation, improvement of endothelial dysfunction, or by directly affecting serum glucose and cholesterol levels by decreasing their absorption or increasing metabolism [152,153]. However, the evidence of these effects mediated by polyphenols is relatively weak in clinical trials because of considerable variability found between individuals. Gut microbial species metabolize polyphenols into simpler ones and aid in increasing their bioavailability, and as the gut microbiota varies between individuals it should be considered as a moderating factor in clinical trials [185]. 

Chen et al. (2016) demonstrated the atherosclerosis attenuating effects of resveratrol by regulating trimethylamine-*N*-oxide (TMAO) synthesis and bile acid metabolism via remodeling of gut microbiota in mice [154]. It was observed that the resveratrol treatment increased bile acid hydrolase activity by increasing the levels of *Lactobacillus* and *Bifidobacterium*, thereby enhancing bile acid deconjugation and fecal excretion in C57BL/6 mice. The authors reported that this was associated with a decrease in ileal content of bile acid, repression of the enterohepatic farnesoid X receptor (FXR)-fibroblast growth factor 15 (FGF15) axis and upregulation of cholesterol 7a-hydroxylase (CYP7A1) expression. Moreover, the results showed decreased levels of TMAO, correlated with inhibiting commensal microbial trimethylamine (TMA) production via gut microbiota remodeling. Additionally, FXR agonists and antibiotics abolished resveratrol induced alterations in FGF15 and TMAO inhibition, respectively.

Zhao et al. (2017) confirmed that a combination of quercetin and resveratrol could restore gut dysbiosis induced by a high-fat diet in mice, and thereby prevent against the pathogenesis of obesity despite low bioavailabilities [155]. The mice were supplemented with a combination of quercetin (30 mg/kg/day) and resveratrol (15 mg/kg/day) by oral gavage. At the end of 10 weeks, results showed that the combination of both flavonoids significantly reduced body weight and visceral adipose tissue (epididymal, perirenal) weight. Serum analysis showed a decrease in serum lipids, reversed serum levels of adiponectin, leptin, and insulin, and attenuation of serum inflammatory markers including IL-6, TNF-α, and monocyte chemotactic protein (MCP)-1. More interestingly the combination was found to modulate gut microbiota, depicted by decreasing *Firmicutes* and the proportion of *Firmicutes* to *Bacteroidetes*. In addition, it also inhibited the relative abundance of *Desulfovibrionaceae*, *Acidaminococcaceae*, *Coriobacteriaceae*, *Bilophila*, *Lachnospiraceae* and its genus *Lachnoclostridium*. Furthermore, the relative abundance of *Bacteroidales*, *Christensenellaceae*, *Akkermansia*, *Ruminococcaceae* and its genus *Ruminococcaceae-UCG-014* and *Ruminococcaceae-UCG-005* was markedly increased, all of which were reported to have positive effects on high-fat diet induced obesity.

Zhang et al. (2020) investigated the effects of xanthohumol derivatives (α,β-dihydroxanthohumol and tetrahydroxanthohumol) on obesity and MS in C57BL/6J male mice treated with 30 mg/kg/day body weight for 13 weeks [186]. Decreases in liver weight, fasting plasma glucose, and insulin level were noted in mice treated with tetrahydroxanthohumol while decreases in plasma leptin level and adipose tissue inflammation was associated with both xanthohumol derivatives. Xanthohumol derivatives were found to decrease intestinal microbiota diversity and abundance, especially *Bacteroidetes* and *Tenericutes*, providing mechanistic insights behind the improvement of obesity and MS. They also altered fecal bile acid metabolism, possibly through modulation of fecal microbiota and microbiome–host interactions. Higher levels of taurine-conjugated bile acids were observed in mice treated with xanthohumol derivatives, which suggested a decrease in deconjugation by the microbiota.

Anhê et al. (2015) investigated the metabolic impact of a polyphenol-rich cranberry extract (*Vaccinium macrocarpon Aiton*) on C57BL/6J mice and determined the effects of cranberry extract on the gut microbiota [187]. The high-fat/high-sucrose (HFHS)-fed mice were treated with vehicle (water) or cranberry extract (200 mg/kg) for 8 weeks through oral gavage. A decrease in body weight gain, visceral obesity, liver weight, triglyceride accumulation in liver, and hepatic oxidative/inflammatory stresses were observed with cranberry extract. On the other hand, cranberry extract improved insulin sensitivity and resistance, glucose-induced hyperinsulinemia, intestinal triglyceride content and intestinal inflammation, and oxidative stress. In addition, cranberry extract administration significantly increased the proportion of the mucin-degrading *Akkermansia* species in the gut microbiota of mice. 

Liu et al. (2017) have reported that grape seed proanthocyanidin extract (GSPE) ameliorated inflammation and adiposity in high-fat diet mice through gut microbial modulation [156]. The mice received GSPE (300 mg/kg body weight/day) for 7 weeks by oral gavage. The results of the study indicate that GSPE significantly improved insulin sensitivity and reduced plasma levels of inflammatory mediators including TNF-α, IL-6, and MCP-1, in addition to ameliorating macrophage infiltration in hepatic and epidydimal fat tissues. 16S rDNA analyses showed a modulation of gut microbial composition and certain bacteria such as *Clostridium XIVa*, *Roseburia*, and *Prevotella* with GSPE supplementation. Interestingly depleting gut microbiota with antibiotic treatment abolished the effects of GSPE supplementation on inflammation and obesity.

## 5. Conclusions

This review attempted to highlight the close link between gut dysbiosis, GI disorders, and MS, as well as the role of bioactive dietary components in influencing numerous pathways associated with the treatment of both disorders. Gut dysbiosis is one of the considerable factors associated with the pathogenesis of GI disorders and MS, by alteration of host’s immune responses and energy homeostasis, which may result in the upstream regulation of inflammatory cascades, insulin resistance, and impairment of the body’s metabolism [49]. Besides gut dysbiosis, researchers also explained the direct link between GI and metabolic disorders. Low-grade chronic inflammatory states in obesity usually facilitate the development and progression of other disorders including IBD [188]. This leads to the statement that prevention of metabolic disorders may also prevent or decrease the frequency of GI disorders like IBD. Despite the availability of a number of therapeutic options, none can provide an ultimate cure with a favorable safety profile. The undesirable effects of drugs have led scientists to consider the use of alternative treatments, including food supplements and functional foods. A review of available scientific literature reveals the health benefits of functional dietary components and their capacity for disease prevention. These have received considerable interest due to their potential nutritional, safety, and protective beneficial effects. In addition to the alteration of other mechanistic pathways, certain dietary components modify gut microbiota, which could provide an alternative approach to reduce a wide range of chronic disorders. In light of the literature available from pre-clinical and clinical studies, the regular consumption of bioactive dietary components in adequate amounts can be said to promote the growth of beneficial bacteria, decrease the inflammatory cascade, regulate intestinal immunity, improve lactose intolerance, enhance the digestive capability of the GI tract, upregulate digestive enzymes, and can improve insulin sensitivity and metabolic pathways. However, the limited available scientific evidence coming from human studies suggests that more in depth clinical trials on these agents in human populations are essential to make these treatments more competitive in the global market of functional foods.

## Figures and Tables

**Table 1:** Benefits of functional dietary components in gastrointestinal disorder in correlation with improvement of gut dysbiosis.

Dietary Components	Potential Benefits in GI Health	References
Dietary fibers	Increase growth and/or activity of beneficial microbes Enhance SCFAs production by intestinal flora Decrease inflammatory cascade Positive impact on constipation and diarrhea Decrease gastrointestinal pain and symptoms of IBS	[62,63,64,65,66]
Prebiotics	Enhance the growth and/or activities of favorable indigenous probiotic bacteria Regulate intestinal immune system Enhance production of SCFAs Improve lactose intolerance Prevent or treat constipation, IBS, and Crohn’s disease	[67,68,69]
Probiotics	Regulate immunity of GI mucosa Decrease gut barrier disruption Inhibit pathogenic microbial growth and activity Improve functional GI symptoms Prevent hepatic pathologies Negatively correlate with *H. pylori* infections Improve lactose intolerance	[70,71,72,73,74]
Polyphenols	Negatively correlate with chronic inflammation of GIT Bidirectional association with gut microbiota Modulates gut microbiota Beneficial effects against *H. pylori* infections Anti-carcinogenic (colon cancer)	[75,76,77]
Spices	Modulate the immune system Negatively regulate inflammatory cascade Reversal of visceral hypersensitivity in IBS Decrease pathogenic bacteria like *H. pylori* Enrichment of SCFAs producing bacteria	[78,79,80]

Irritable bowel syndrome (IBS); gastrointestinal mucosa (GI mucosa); *Helicobacter pylori* (*H. pylori*); short chain fatty acids (SCFAs); gastrointestinal tract (GIT).

**Table 2:** Benefits of functional dietary components in metabolic syndrome in correlation with improvements in gut dysbiosis.

Dietary Components	Potential Benefits in MS	References
Dietary fibers	Improve insulin sensitivity Decrease glucose concentration Decrease TC, LDL, and TG Body weight reduction Increase fatty acid oxidation Increase level of SCFAs Regulate inflammatory biomarkers Improve endothelial dysfunction	[133,134]
Prebiotics	Enhance the growth and/or activity of probiotic species Improve insulin sensitivity Increase glucose tolerance Decrease insulin resistance Upregulate expression of anti-inflammatory cytokines Increase lipolysis and decrease adipogenesis Decrease inflammatory mediators	[135,136,137]
Probiotics	Modulation of gut microbiota Increase level of SCFAs Upregulate PPARα–FGF21 signaling Body weight reduction Improve insulin resistance and glucose intolerance Improve intestinal barrier function Protect pancreas against apoptosis Increase insulin secretion through enhanced expression of GLP-1 Decrease LDL concentration	[138,139,140,141]
Short chain fatty acids	Improve gut dysbiosis Positively regulate host metabolism Upregulate expression of GPR 43 Decrease systemic inflammatory responses Increase abundance of mucin-foraging bacteria (*Akkermansia muciniphila*) Increase lipolysis through increasing ANGPTL4 expression in human epithelial cells	[142,143,144,145]
Monounsaturated fatty acids (oleic acid)	Improve gut dysbiosis by increasing abundance of bifidobacteria Improve insulin sensitivity	[146,147]
Polyunsaturated fatty acids (omega-3 fatty acids)	Improve gut dysbiosis by reducing bacterial overgrowth (*H. pylori*, *Pseudomonas,* and Firmicutes) Recruit regulatory T cells and regulate inflammatory responses Improve metabolic endotoxemia Increase insulin sensitivity by upregulation of cell surface expression of GLUT4 Increase insulin secretion by activating GPR 40	[148,149,150,151]
Polyphenols	Improve insulin signaling Decrease blood glucose and cholesterol levels Reduce body weight and visceral adipose tissues weight Gut microbial modulation Decrease serum level of lipids Improve endothelial dysfunction Decrease oxidative damage and inflammatory mediators Decrease ileal content of bile acid Repression of the enterohepatic FXR–FGF15 axis Upregulate CYP7A1 expression	[152,153,154,155,156]

Total cholesterol (TC); low density lipoprotein (LDL); triglycerides (TG); short chain fatty acids (SCFAs); peroxisome proliferator activated receptor alpha (PPARα); fibroblast growth factor 21 (FGF21); glucagon like peptide-1 (GLP-1); G-protein-coupled receptor 43 (GPR 43); angiopoietin-like protein-4 (ANGPTL4); glucose transporter type 4 (GLUT4); G-protein coupled receptor 40 (GPR 40); farnesoid X receptor (FXR); fibroblast growth factor 15 (FGF15); cholesterol 7a-hydroxylase (CYP7A1).

## Abbreviations

ADA	American Diabetes Association
ANGPTL4	Angiopoietin-like protein-4
BMI	Body mass index
CD	Crohn’s disease
CD8	Cluster of differentiation 8
CFU	Colony forming units
COX-2	Cyclooxygenase-2
CYP7A1	Cholesterol 7a-hydroxylase
DSS	Dextran sulfate sodium
EGCG	Epigallocatechin-3- gallate
FDA	Food and Drug Administration
FGF15	Fibroblast growth factor 15
FGF21	Fibroblast growth factor 21
FFA	Free fatty acids
FOS	Fructo-oligosaccharide
FXR	Farnesoid X receptor
GABA	Gamma-aminobutyric acid
GERD	Gastrointestinal reflux disease
GIT	Gastrointestinal tract
GLP-1	Glucagon like peptide-1
GLUT4	Glucose transporter type 4
GOS	Galacto-oligosaccharide
GPR 40	G-protein coupled receptor 40
GSPE	Grape seed proanthocyanidin extract
HDL	High density lipoprotein
HOMA-β	Homeostatic model assessment for β cell function
HOMA-IR	Homeostatic model assessment for insulin resistance
HSHF	High-sucrose high-fat diet
IBD	Inflammatory bowel diseases
IBS	Irritable bowel syndrome
IDF	International Diabetes Federation
IFN-γ	Interferon-gamma
IL	Interleukin
JNK	Jun N-terminal kinase
KYN	Kynurenine
LDL	Low density lipoprotein
LPS	Lipopolysaccharide
MAPK	Mitogen activated protein kinase
MCP-1	Monocyte chemotactic protein-1
MIC	Minimum inhibitory concentration
MS	Metabolic syndrome
NAFLD	Nonalcoholic fatty liver disease
NF-kβ	Nuclear factor-kappa Beta
NSAIDs	Nonsteroidal anti-inflammatory drugs
NSP	Non-starch polysaccharides
PGE2	Prostaglandin E2
PPARα	Peroxisome proliferator activated receptor alpha
PUFAs	Polyunsaturated fatty acids
RAAS	Renin-angiotensin-aldosterone system
SCFAs	Short-chain fatty acids
T2DM	Type 2 diabetes mellitus
TC	Total cholesterol
TG	Triglycerides
TLR	Toll-like receptor
TMAO	Trimethylamine-*N*-oxide
TNF-α	Tumor necrosis factor alpha
TRP	Tryptophan
UC	Ulcerative colitis
WHO	World Health Organization

## References

[1] Oshima T., Miwa H. (2015). Epidemiology of functional gastrointestinal disorders in Japan and in the world. J. Neurogastroenterol. Motil..

[2] Drossman D.A. (2016). Functional gastrointestinal disorders: History, pathophysiology, clinical features, and Rome IV. Gastroenterology.

[3] Lichtenstein G.R., Loftus E.V., Isaacs K.L., Regueiro M.D., Gerson L.B., Sands B.E. (2018). ACG clinical guideline: Management of Crohn’s disease in adults. Am. J. Gastroenterol..

[4] Danese S., Fiorino G., Peyrin-Biroulet L. (2020). Positioning therapies in ulcerative colitis. Clin. Gastroenterol. Hepatol..

[5] Barbara G., Scaioli E., Barbaro M.R., Biagi E., Laghi L., Cremon C., Marasco G., Colecchia A., Picone G., Salfi N. (2017). Gut microbiota, metabolome and immune signatures in patients with uncomplicated diverticular disease. Gut.

[6] Holtmann G., Shah A., Morrison M. (2017). Pathophysiology of functional gastrointestinal disorders: A holistic overview. Dig. Dis..

[7] Philpott H., Nandurkar S., Lubel J., Gibson P.R. (2014). Republished: Drug-induced gastrointestinal disorders. Postgrad. Med. J..

[8] Francino M. (2016). Antibiotics and the human gut microbiome: Dysbioses and accumulation of resistances. Front. Microbiol..

[9] Scarborough P., Bhatnagar P., Wickramasinghe K.K., Allender S., Foster C., Rayner M. (2011). The economic burden of ill health due to diet, physical inactivity, smoking, alcohol and obesity in the UK: An update to 2006–07 NHS costs. J. Public Health.

[10] Sachs G., Scott D.R. (2012). Helicobacter pylori: Eradication or preservation. F1000 Med. Rep..

[11] Ananthakrishnan A.N., Xavier R.J. (2020). Gastrointestinal diseases. Hunter’s Tropical Medicine and Emerging Infectious Diseases.

[12] McQuaid K.R., Katzung B.G. (2012). Drugs used in the treatment of gastrointestinal diseases. Basic and Clinical Pharmacology.

[13] Gangarosa L.M., Seibert D.G., Craig C.R., Stitzel R.E. (2004). Drugs used in gastrointestinal disorders. Modern Pharmacology with Clinical Applications.

[14] Hindryckx P., Novak G., Bonovas S., Peyrin-Biroulet L., Danese S. (2017). Infection risk with biologic therapy in patients with inflammatory bowel disease. Clin. Pharmacol. Ther..

[15] Parfitt J.R., Driman D.K. (2007). Pathological effects of drugs on the gastrointestinal tract: A review. Hum. Pathol..

[16] El-Serag H.B., Sweet S., Winchester C.C., Dent J. (2014). Update on the epidemiology of gastro-oesophageal reflux disease: A systematic review. Gut.

[17] Ford A.C., Marwaha A., Sood R., Moayyedi P. (2015). Global prevalence of, and risk factors for, uninvestigated dyspepsia: A meta-analysis. Gut.

[18] Scallan E., Griffin P.M., Angulo F.J., Tauxe R.V., Hoekstra R.M. (2011). Foodborne illness acquired in the United States—Unspecified agents. Emerg. Infect. Dis..

[19] Scallan E., Hoekstra R.M., Angulo F.J., Tauxe R.V., Widdowson M.-A., Roy S.L., Jones J.L., Griffin P.M. (2011). Foodborne illness acquired in the United States—Major pathogens. Emerg. Infect. Dis..

[20] Wald A., Scarpignato C., Mueller-Lissner S., Kamm M., Hinkel U., Helfrich I., Schuijt C., Mandel K. (2008). A multinational survey of prevalence and patterns of laxative use among adults with self-defined constipation. Aliment. Pharmacol. Ther..

[21] Nyrop K., Palsson O., Levy R., Korff M.V., Feld A., Turner M., Whitehead W. (2007). Costs of health care for irritable bowel syndrome, chronic constipation, functional diarrhoea and functional abdominal pain. Aliment. Pharmacol. Ther..

[22] Oświęcimska J., Szymlak A., Roczniak W., Girczys-Połedniok K., Kwiecień J. (2017). New insights into the pathogenesis and treatment of irritable bowel syndrome. Adv. Med. Sci..

[23] Aguirre J.E., Winston J., Sarna S.K. (2017). Neonatal immune challenge followed by adult immune challenge induces epigenetic-susceptibility to aggravated visceral hypersensitivity. Neurogastroenterol. Motil..

[24] Toro-Martín D., Arsenault B.J., Després J.-P., Vohl M.-C. (2017). Precision nutrition: A review of personalized nutritional approaches for the prevention and management of metabolic syndrome. Nutrients.

[25] Engin A. (2017). The definition and prevalence of obesity and metabolic syndrome. Obesity and Lipotoxicity.

[26] Reaven G.M. (1988). Banting lecture 1988. Role of insulin resistance in human disease. Diabetes.

[27] Mongraw-Chaffin M., Foster M.C., Anderson C.A., Burke G.L., Haq N., Kalyani R.R., Ouyang P., Sibley C.T., Tracy R., Woodward M. (2018). Metabolically healthy obesity, transition to metabolic syndrome, and cardiovascular risk. J. Am. Coll. Cardiol..

[28] Åberg F., Helenius-Hietala J., Puukka P., Färkkilä M., Jula A. (2018). Interaction between alcohol consumption and metabolic syndrome in predicting severe liver disease in the general population. Hepatology.

[29] Alberti K., Eckel R.H., Grundy S.M., Zimmet P.Z., Cleeman J.I., Donato K.A., Fruchart J.-C., James W.P.T., Loria C.M., Smith S.C. (2009). Harmonizing the metabolic syndrome: A joint interim statement of the international diabetes federation task force on epidemiology and prevention; national heart, lung, and blood institute; American heart association; world heart federation; international atherosclerosis society; and international association for the study of obesity. Circulation.

[30] Sadeghi M. (2010). The metabolic syndrome. Arya Atheroscler..

[31] Vague J. (1947). Sexual differentiation, a factor affecting the forms of obesity. La Presse Médicale.

[32] Alberti K.G.M.M., Zimmet P.Z. (1998). Definition, diagnosis and classification of diabetes mellitus and its complications. Part 1: Diagnosis and classification of diabetes mellitus. Provisional report of a WHO consultation. Diabet. Med..

[33] Balkau B. (1999). Comment on the provisional report from the WHO consultation. European Group for the Study of Insulin Resistance (EGIR). Diabet. Med..

[34] Zimmet P., Alberti K., Shaw J. (2005). International Diabetes Federation: The IDF consensus worldwide definition of the metabolic syndrome. Diabetes Voice.

[35] Lipińska A., Koczaj-Bremer M., Jankowski K., Kaźmierczak A., Ciurzyński M., Ou-Pokrzewińska A., Mikocka E., Lewandowski Z., Demkow U., Pruszczyk P. (2014). Does family history of metabolic syndrome affect the metabolic profile phenotype in young healthy individuals?. Diabetol. Metab. Syndr..

[36] Sun K., Liu J., Ning G. (2012). Active smoking and risk of metabolic syndrome: A meta-analysis of prospective studies. PLoS ONE.

[37] Fan A.Z., Russell M., Naimi T., Li Y., Liao Y., Jiles R., Mokdad A.H. (2008). Patterns of alcohol consumption and the metabolic syndrome. J. Clin. Endocrinol. Metab..

[38] Park Y.-W., Zhu S., Palaniappan L., Heshka S., Carnethon M.R., Heymsfield S.B. (2003). The metabolic syndrome: Prevalence and associated risk factor findings in the US population from the Third National Health and Nutrition Examination Survey, 1988–1994. Arch. Intern. Med..

[39] Gennuso K.P., Gangnon R.E., Thraen-Borowski K.M., Colbert L.H. (2015). Dose–response relationships between sedentary behaviour and the metabolic syndrome and its components. Diabetologia.

[40] Lutsey P.L., Steffen L.M., Stevens J. (2008). Dietary intake and the development of the metabolic syndrome. Circulation.

[41] Lamberti J.S., Olson D., Crilly J.F., Olivares T., Williams G.C., Tu X., Tang W., Wiener K., Dvorin S., Dietz M.B. (2006). Prevalence of the metabolic syndrome among patients receiving clozapine. Am. J. Psychiatry.

[42] Lim S., Eckel R.H. (2014). Pharmacological treatment and therapeutic perspectives of metabolic syndrome. Rev. Endocr. Metab. Disord..

[43] Rask Larsen J., Dima L., Correll C.U., Manu P. (2018). The pharmacological management of metabolic syndrome. Expert Rev. Clin. Pharmacol..

[44] Prasad H., Ryan D.A., Celzo M.F., Stapleton D. (2012). Metabolic syndrome: Definition and therapeutic implications. Postgrad. Med..

[45] Stern M.P., Williams K., González-Villalpando C., Hunt K.J., Haffner S.M. (2004). Does the metabolic syndrome improve identification of individuals at risk of type 2 diabetes and/or cardiovascular disease?. Diabetes Care.

[46] WHO Obesity and Overweight. https://www.who.int/news-room/fact-sheets/detail/obesity-and-overweight.

[47] WHO Hypertension. https://www.who.int/news-room/fact-sheets/detail/hypertension.

[48] WHO Diabetes. https://www.who.int/news-room/fact-sheets/detail/diabetes.

[49] Carding S., Verbeke K., Vipond D.T., Corfe B.M., Owen L.J. (2015). Dysbiosis of the gut microbiota in disease. Microb. Ecol. Health Dis..

[50] Tang T.W., Chen H.-C., Chen C.-Y., Yen C.Y., Lin C.-J., Prajnamitra R.P., Chen L.-L., Ruan S.-C., Lin J.-H., Lin P.-J. (2019). Loss of gut microbiota alters immune system composition and cripples postinfarction cardiac repair. Circulation.

[51] Hur K.Y., Lee M.-S. (2015). Gut microbiota and metabolic disorders. Diabetes Metab. J..

[52] Terrapon N., Henrissat B. (2014). How do gut microbes break down dietary fiber?. Trends Biochem. Sci..

[53] Prasad K.N., Bondy S.C. (2019). Dietary fibers and their fermented short-chain fatty acids in prevention of human diseases. Bioact. Carbohydr. Diet. Fibre.

[54] Barbara G., Feinle-Bisset C., Ghoshal U.C., Santos J., Vanner S.J., Vergnolle N., Zoetendal E.G., Quigley E.M. (2016). The intestinal microenvironment and functional gastrointestinal disorders. Gastroenterology.

[55] Kannampalli P., Shaker R., Sengupta J.N. (2011). Colonic butyrate-algesic or analgesic?. Neurogastroenterol. Motil..

[56] Tana C., Umesaki Y., Imaoka A., Handa T., Kanazawa M., Fukudo S. (2010). Altered profiles of intestinal microbiota and organic acids may be the origin of symptoms in irritable bowel syndrome. Neurogastroenterol. Motil..

[57] Talukder J., Gupta R.C., Srivastava A., Lall R. (2019). Nutraceuticals in gastrointestinal conditions. Nutraceuticals in Veterinary Medicine.

[58] Myasoedova E., Talley N.J., Manek N.J., Crowson C.S. (2011). Prevalence and risk factors of gastrointestinal disorders in patients with rheumatoid arthritis: Results from a population-based survey in Olmsted County, Minnesota. Gastroenterol. Res. Pract..

[59] Chuah K.-H., Mahadeva S. (2018). Cultural factors influencing functional gastrointestinal disorders in the east. J. Neurogastroenterol. Motil..

[60] Ribaldone D.G., Pellicano R., Actis G.C. (2019). Inflammation in gastrointestinal disorders: Prevalent socioeconomic factors. Clin. Exp. Gastroenterol..

[61] Gul K., Singh A., Jabeen R. (2016). Nutraceuticals and functional foods: The foods for the future world. Crit. Rev. Food Sci. Nutr..

[62] Parada Venegas D., De la Fuente M.K., Landskron G., González M.J., Quera R., Dijkstra G., Harmsen H.J., Faber K.N., Hermoso M.A. (2019). Short chain fatty acids (SCFAs)-mediated gut epithelial and immune regulation and its relevance for inflammatory bowel diseases. Front. Immunol..

[63] Den Besten G., van Eunen K., Groen A.K., Venema K., Reijngoud D.-J., Bakker B.M. (2013). The role of short-chain fatty acids in the interplay between diet, gut microbiota, and host energy metabolism. J. Lipid Res..

[64] El-Salhy M., Ystad S.O., Mazzawi T., Gundersen D. (2017). Dietary fiber in irritable bowel syndrome. Int. J. Mol. Med..

[65] Zhang M., Zhou Q., Dorfman R.G., Huang X., Fan T., Zhang H., Zhang J., Yu C. (2016). Butyrate inhibits interleukin-17 and generates Tregs to ameliorate colorectal colitis in rats. BMC Gastroenterol..

[66] Velazquez O.C., Lederer H.M., Rombeau J.L. (1997). Butyrate and the colonocyte: Production, absorption, metabolism, and therapeutic implications. Dietary Fiber in Health and Disease.

[67] Al-Sheraji S.H., Ismail A., Manap M.Y., Mustafa S., Yusof R.M., Hassan F.A. (2013). Prebiotics as functional foods: A review. J. Funct. Foods.

[68] Wilson B., Whelan K. (2017). Prebiotic inulin-type fructans and galacto-oligosaccharides: Definition, specificity, function, and application in gastrointestinal disorders. J. Gastroenterol. Hepatol..

[69] Eslamparast T., Zamani F., Hekmatdoost A., Sharafkhah M., Eghtesad S., Malekzadeh R., Poustchi H. (2014). Effects of synbiotic supplementation on insulin resistance in subjects with the metabolic syndrome: A randomised, double-blind, placebo-controlled pilot study. Br. J. Nutr..

[70] Bron P.A., Kleerebezem M., Brummer R.-J., Cani P.D., Mercenier A., MacDonald T.T., Garcia-Ródenas C.L., Wells J.M. (2017). Can probiotics modulate human disease by impacting intestinal barrier function?. Br. J. Nutr..

[71] Kang H.-J., Im S.-H. (2015). Probiotics as an immune modulator. J. Nutr. Sci. Vitaminol..

[72] Srinarong C., Siramolpiwat S., Wongcha-um A., Mahachai V., Vilaichone R.-K. (2014). Improved eradication rate of standard triple therapy by adding bismuth and probiotic supplement for Helicobacter pylori treatment in Thailand. Asian Pac. J. Cancer Prev..

[73] Almeida C.C., Lorena S.L.S., Pavan C.R., Akasaka H.M.I., Mesquita M.A. (2012). Beneficial effects of long-term consumption of a probiotic combination of Lactobacillus casei Shirota and Bifidobacterium breve Yakult may persist after suspension of therapy in lactose-intolerant patients. Nutr. Clin. Pract..

[74] Xue L., He J., Gao N., Lu X., Li M., Wu X., Liu Z., Jin Y., Liu J., Xu J. (2017). Probiotics may delay the progression of nonalcoholic fatty liver disease by restoring the gut microbiota structure and improving intestinal endotoxemia. Sci. Rep..

[75] Larrosa M., Yañéz-Gascón M.A.J., Selma M.A.V., Gonzalez-Sarrias A., Toti S., Cerón J.J.N., Tomas-Barberan F., Dolara P., Espín J.C. (2009). Effect of a low dose of dietary resveratrol on colon microbiota, inflammation and tissue damage in a DSS-induced colitis rat model. J. Agric. Food Chem..

[76] Cardona F., Andrés-Lacueva C., Tulipani S., Tinahones F.J., Queipo-Ortuño M.I. (2013). Benefits of polyphenols on gut microbiota and implications in human health. J. Nutr. Biochem..

[77] Lee K.M., Yeo M., Choue J.S., Jin J.H., Park S.J., Cheong J.Y., Lee K.J., Kim J.H., Hahm K.B. (2004). Protective mechanism of epigallocatechin-3-gallate against Helicobacter pylori-induced gastric epithelial cytotoxicity via the blockage of TLR-4 signaling. Helicobacter.

[78] Ohno M., Nishida A., Sugitani Y., Nishino K., Inatomi O., Sugimoto M., Kawahara M., Andoh A. (2017). Nanoparticle curcumin ameliorates experimental colitis via modulation of gut microbiota and induction of regulatory T cells. PLoS ONE.

[79] Botschuijver S., Welting O., Levin E., Maria-Ferreira D., Koch E., Montijn R., Seppen J., Hakvoort T., Schuren F., De Jonge W. (2018). Reversal of visceral hypersensitivity in rat by Menthacarin^®^, a proprietary combination of essential oils from peppermint and caraway, coincides with mycobiome modulation. Neurogastroenterol. Motil..

[80] Li A.-L., Ni W.-W., Zhang Q.-M., Li Y., Zhang X., Wu H.-Y., Du P., Hou J.-C., Zhang Y. (2020). Effect of cinnamon essential oil on gut microbiota in the mouse model of dextran sodium sulfate-induced colitis. Microbiol. Immunol..

[81] Longe J.L. (2008). The Gale Encyclopedia of Diets: A Guide to Health and Nutrition.

[82] Rana V., Bachheti R.K., Chand T., Barman A. (2011). Dietary fibre and human health. Int. J. Food Saf. Nutr. Public Health.

[83] Anderson J.W., Baird P., Davis R.H., Ferreri S., Knudtson M., Koraym A., Waters V., Williams C.L. (2009). Health benefits of dietary fiber. Nutr. Rev..

[84] Asp N.G., Johansson C.G., Hallmer H., Siljestroem M. (1983). Rapid enzymic assay of insoluble and soluble dietary fiber. J. Agric. Food Chem..

[85] Johnson I., Caballero B. (2005). Fiber: Physiological and functional effects. Encyclopedia of Human Nutrition.

[86] Camilleri M. (2001). Management of the irritable bowel syndrome. Gastroenterology.

[87] Camilleri M., Heading R., Thompson W. (2002). Consensus report: Clinical perspectives, mechanisms, diagnosis and management of irritable bowel syndrome. Aliment. Pharmacol. Ther..

[88] Hamer H.M., Jonkers D., Venema K., Vanhoutvin S., Troost F., Brummer R.J. (2008). The role of butyrate on colonic function. Aliment. Pharmacol. Ther..

[89] Allan E., Winter S., Light A., Allan A. (1996). Mucosal enzyme activity for butyrate oxidation; no defect in patients with ulcerative colitis. Gut.

[90] Floch M.H. (2018). The role of prebiotics and probiotics in gastrointestinal disease. Gastroenterol. Clin..

[91] Hord N.G. (2008). Eukaryotic-microbiota crosstalk: Potential mechanisms for health benefits of prebiotics and probiotics. Annu. Rev. Nutr..

[92] Gibson G.R., Probert H.M., Van Loo J., Rastall R.A., Roberfroid M.B. (2004). Dietary modulation of the human colonic microbiota: Updating the concept of prebiotics. Nutr. Res. Rev..

[93] Davani-Davari D., Negahdaripour M., Karimzadeh I., Seifan M., Mohkam M., Masoumi S.J., Berenjian A., Ghasemi Y. (2019). Prebiotics: Definition, types, sources, mechanisms, and clinical applications. Foods.

[94] Verna E.C., Lucak S. (2010). Use of probiotics in gastrointestinal disorders: What to recommend?. Ther. Adv. Gastroenterol..

[95] Mishra S.S., Behera P.K., Kar B., Ray R.C. (2018). Advances in probiotics, prebiotics and nutraceuticals. Innovations in Technologies for Fermented Food and Beverage Industries.

[96] Suvarna V., Boby V. (2005). Probiotics in human health: A current assessment. Curr. Sci..

[97] Chugh B., Kamal-Eldin A. (2020). Bioactive compounds produced by probiotics in food products. Curr. Opin. Food Sci..

[98] Delgado S., Sánchez B., Margolles A., Ruas-Madiedo P., Ruiz L. (2020). Molecules produced by probiotics and intestinal microorganisms with immunomodulatory activity. Nutrients.

[99] Krishna Rao R., Samak G. (2013). Protection and restitution of gut barrier by probiotics: Nutritional and clinical implications. Curr. Nutr. Food Sci..

[100] Sreeja V., Prajapati J.B. (2013). Probiotic formulations: Application and status as pharmaceuticals—A review. Probiotics Antimicrob. Proteins.

[101] Jesus A.L.T., Fernandes M.S., Kamimura B.A., Prado-Silva L., Silva R., Esmerino E.A., Cruz A.G., Sant’Ana A.S. (2016). Growth potential of Listeria monocytogenes in probiotic cottage cheese formulations with reduced sodium content. Food Res. Int..

[102] Chen C.-C., Kong M.-S., Lai M.-W., Chao H.-C., Chang K.-W., Chen S.-Y., Huang Y.-C., Chiu C.-H., Li W.-C., Lin P.-Y. (2010). Probiotics have clinical, microbiologic, and immunologic efficacy in acute infectious diarrhea. Pediatr. Infect. Dis. J..

[103] Fooladi A.A.I., Hosseini H.M., Nourani M.R., Khani S., Alavian S.M. (2013). Probiotic as a novel treatment strategy against liver disease. Zahedan J. Res. Med Sci..

[104] Indrio F., Riezzo G., Raimondi F., Bisceglia M., Filannino A., Cavallo L., Francavilla R. (2011). Lactobacillus reuteri accelerates gastric emptying and improves regurgitation in infants. Eur. J. Clin. Investig..

[105] Cammarota G., Ianiro G., Cianci R., Bibbò S., Gasbarrini A., Currò D. (2015). The involvement of gut microbiota in inflammatory bowel disease pathogenesis: Potential for therapy. Pharmacol. Ther..

[106] Waller P.A., Gopal P.K., Leyer G.J., Ouwehand A.C., Reifer C., Stewart M.E., Miller L.E. (2011). Dose-response effect of Bifidobacterium lactis HN019 on whole gut transit time and functional gastrointestinal symptoms in adults. Scand. J. Gastroenterol..

[107] Vinson J.A., Su X., Zubik L., Bose P. (2001). Phenol antioxidant quantity and quality in foods: Fruits. J. Agric. Food Chem..

[108] Kabera J.N., Semana E., Mussa A.R., He X. (2014). Plant secondary metabolites: Biosynthesis, classification, function and pharmacological properties. J. Pharm. Pharmacol..

[109] Hollman P.C.H., Katan M.B. (1999). Dietary flavonoids: Intake, health effects and bioavailability. Food Chem. Toxicol..

[110] Andrew R., Izzo A.A. (2017). Principles of pharmacological research of nutraceuticals. Br. J. Pharmacol..

[111] Khan H., Ullah H., Tundis R., Belwal T., Devkota H.P., Daglia M., Cetin Z., Saygili E.I., da Graça Campos M., Capanoglu E. (2020). Dietary flavonoids in the management of huntington’s disease: Mechanism and clinical perspective. eFood.

[112] Scalbert A., Johnson I.T., Saltmarsh M. (2005). Polyphenols: Antioxidants and beyond. Am. J. Clin. Nutr..

[113] Fraga C.G., Galleano M., Verstraeten S.V., Oteiza P.I. (2010). Basic biochemical mechanisms behind the health benefits of polyphenols. Mol. Asp. Med..

[114] González R., Ballester I., López-Posadas R., Suárez M., Zarzuelo A., Martinez-Augustin O., Medina F.S.D. (2011). Effects of flavonoids and other polyphenols on inflammation. Crit. Rev. Food Sci. Nutr..

[115] Scheepens A., Tan K., Paxton J.W. (2010). Improving the oral bioavailability of beneficial polyphenols through designed synergies. Genes Nutr..

[116] Frolinger T., Sims S., Smith C., Wang J., Cheng H., Faith J., Ho L., Hao K., Pasinetti G.M. (2019). The gut microbiota composition affects dietary polyphenols-mediated cognitive resilience in mice by modulating the bioavailability of phenolic acids. Sci. Rep..

[117] Saha P., San Yeoh B., Singh R., Chandrasekar B., Vemula P.K., Haribabu B., Vijay-Kumar M., Jala V.R. (2016). Gut microbiota conversion of dietary ellagic acid into bioactive phytoceutical urolithin a inhibits heme peroxidases. PLoS ONE.

[118] Selma M.V., Espin J.C., Tomas-Barberan F.A. (2009). Interaction between phenolics and gut microbiota: Role in human health. J. Agric. Food Chem..

[119] Singh Y., Zhang S., Al-Maghout T., Cao I., Pelzl L., Salker M., Veldhoen M., Cheng A., Lang F. (2019). Gut bacterial metabolite Urolithin A (UA) mitigates Ca^2+^ entry in T cells by regulating miR-10a-5p. Front. Immunol..

[120] Mena P., Dall’Asta M., Calani L., Brighenti F., Del Rio D. (2017). Gastrointestinal stability of urolithins: An in vitro approach. Eur. J. Nutr..

[121] Selma M.V., Beltrán D., Luna M.C., Romo-Vaquero M., García-Villalba R., Mira A., Espín J.C., Tomás-Barberán F.A. (2017). Isolation of human intestinal bacteria capable of producing the bioactive metabolite isourolithin a from ellagic acid. Front. Microbiol..

[122] Meng C., Bai C., Brown T.D., Hood L.E., Tian Q. (2018). Human gut microbiota and gastrointestinal cancer. Genom. Proteom. Bioinform..

[123] Allsopp P., Possemiers S., Campbell D., Gill C., Rowland I. (2013). A comparison of the anticancer properties of isoxanthohumol and 8-prenylnaringenin using in vitro models of colon cancer. Biofactors.

[124] Gupta M. (2010). Pharmacological properties and traditional therapeutic uses of important Indian spices: A review. Int. J. Food Prop..

[125] Singh V.K., Yadav P., Tadigoppula N. (2014). Recent advances in the synthesis, chemical transformations and pharmacological studies of some important dietary spice’s constituents. Chem. Biol. Interface.

[126] Kochhar K. (2008). Dietary spices in health and diseases: I. Indian J. Physiol. Pharmacol..

[127] Platel K., Srinivasan K. (2004). Digestive stimulant action of spices: A myth or reality?. Indian J. Med Res..

[128] Shen L., Ji H.-F. (2016). Intestinal microbiota and metabolic diseases: Pharmacological Implications. Trends Pharmacol. Sci..

[129] Cao S.-Y., Zhao C.-N., Xu X.-Y., Tang G.-Y., Corke H., Gan R.-Y., Li H.-B. (2019). Dietary plants, gut microbiota, and obesity: Effects and mechanisms. Trends Food Sci. Technol..

[130] Ríos-Hoyo A., Cortés M.J., Rios-Ontiveros H., Meaney E., Ceballos G., Gutierrez-Salmean G. (2014). Obesity, metabolic syndrome, and dietary therapeutical approaches with a special focus on nutraceuticals (polyphenols): A mini-review. Int. J. Vitam. Nutr. Res..

[131] Davì G., Santilli F., Patrono C. (2010). Nutraceuticals in diabetes and metabolic syndrome. Cardiovasc. Ther..

[132] Chakraborty R., Das L., Bagchi D., Preuss H.G., Swaroop A. (2015). Nutraceuticals and their role in human health: A review. Nutraceuticals and Functional Foods in Human Health and Disease Prevention.

[133] Kjølbæk L., Benítez-Páez A., Del Pulgar E.M.G., Brahe L.K., Liebisch G., Matysik S., Rampelli S., Vermeiren J., Brigidi P., Larsen L.H. (2020). Arabinoxylan oligosaccharides and polyunsaturated fatty acid effects on gut microbiota and metabolic markers in overweight individuals with signs of metabolic syndrome: A randomized cross-over trial. Clin. Nutr..

[134] Li K., Zhang L., Xue J., Yang X., Dong X., Sha L., Lei H., Zhang X., Zhu L., Wang Z. (2019). Dietary inulin alleviates diverse stages of type 2 diabetes mellitus via anti-inflammation and modulating gut microbiota in db/db mice. Food Funct..

[135] Mäkeläinen H., Saarinen M., Stowell J., Rautonen N., Ouwehand A. (2010). Xylo-oligosaccharides and lactitol promote the growth of Bifidobacterium lactis and Lactobacillus species in pure cultures. Benef. Microbes.

[136] de Cossío L.F., Fourrier C., Sauvant J., Everard A., Capuron L., Cani P.D., Layé S., Castanon N. (2017). Impact of prebiotics on metabolic and behavioral alterations in a mouse model of metabolic syndrome. Brain Behav. Immun..

[137] Everard A., Lazarevic V., Derrien M., Girard M., Muccioli G.G., Neyrinck A.M., Possemiers S., Van Holle A., François P., de Vos W.M. (2011). Responses of gut microbiota and glucose and lipid metabolism to prebiotics in genetic obese and diet-induced leptin-resistant mice. Diabetes.

[138] Sroka-Oleksiak A., Młodzińska A., Bulanda M., Salamon D., Major P., Stanek M., Gosiewski T. (2020). Metagenomic analysis of duodenal microbiota reveals a potential biomarker of dysbiosis in the course of obesity and type 2 diabetes: A pilot study. J. Clin. Med..

[139] Liu Q., Liu Y., Li F., Gu Z., Liu M., Shao T., Zhang L., Zhou G., Pan C., He L. (2020). Probiotic culture supernatant improves metabolic function through FGF21-adiponectin pathway in mice. J. Nutr. Biochem..

[140] Wang Y., Dilidaxi D., Wu Y., Sailike J., Sun X., Nabi X.-H. (2020). Composite probiotics alleviate type 2 diabetes by regulating intestinal microbiota and inducing GLP-1 secretion in db/db mice. Biomed. Pharmacother..

[141] Michael D., Jack A., Masetti G., Davies T., Loxley K., Kerry-Smith J., Plummer J., Marchesi J., Mullish B., McDonald J. (2020). A randomised controlled study shows supplementation of overweight and obese adults with lactobacilli and bifidobacteria reduces bodyweight and improves well-being. Sci. Rep..

[142] Marette A., Jobin C. (2015). SCFAs take a toll en route to metabolic syndrome. Cell Metab..

[143] Kimura I., Ozawa K., Inoue D., Imamura T., Kimura K., Maeda T., Terasawa K., Kashihara D., Hirano K., Tani T. (2013). The gut microbiota suppresses insulin-mediated fat accumulation via the short-chain fatty acid receptor GPR43. Nat. Commun..

[144] Everard A., Lazarevic V., Gaïa N., Johansson M., Ståhlman M., Backhed F., Delzenne N.M., Schrenzel J., François P., Cani P.D. (2014). Microbiome of prebiotic-treated mice reveals novel targets involved in host response during obesity. ISME J..

[145] Korecka A., de Wouters T., Cultrone A., Lapaque N., Pettersson S., Doré J., Blottière H.M., Arulampalam V. (2013). ANGPTL4 expression induced by butyrate and rosiglitazone in human intestinal epithelial cells utilizes independent pathways. Am. J. Physiol. Liver Physiol..

[146] Mujico J.R., Baccan G.C., Gheorghe A., Díaz L.E., Marcos A. (2013). Changes in gut microbiota due to supplemented fatty acids in diet-induced obese mice. Br. J. Nutr..

[147] Kien C.L., Bunn J.Y., Poynter M.E., Stevens R., Bain J., Ikayeva O., Fukagawa N.K., Champagne C.M., Crain K.I., Koves T.R. (2013). A lipidomics analysis of the relationship between dietary fatty acid composition and insulin sensitivity in young adults. Diabetes.

[148] Ghosh S., Molcan E., DeCoffe D., Dai C., Gibson D.L. (2013). Diets rich in n-6 PUFA induce intestinal microbial dysbiosis in aged mice. Br. J. Nutr..

[149] Yu H.-N., Zhu J., Pan W.-S., Shen S.-R., Shan W.-G., Das U.N. (2014). Effects of fish oil with a high content of n-3 polyunsaturated fatty acids on mouse gut microbiota. Arch. Med. Res..

[150] Talukdar S., Bae E.J., Imamura T., Morinaga H., Fan W., Li P., Lu W.J., Watkins S.M., Olefsky J.M. (2010). GPR120 is an omega-3 fatty acid receptor mediating potent anti-inflammatory and insulin-sensitizing effects. Cell.

[151] Itoh Y., Kawamata Y., Harada M., Kobayashi M., Fujii R., Fukusumi S., Ogi K., Hosoya M., Tanaka Y., Uejima H. (2003). Free fatty acids regulate insulin secretion from pancreatic β cells through GPR40. Nature.

[152] Cao H., Ou J., Chen L., Zhang Y., Szkudelski T., Delmas D., Daglia M., Xiao J. (2019). Dietary polyphenols and type 2 diabetes: Human study and clinical trial. Crit. Rev. Food Sci. Nutr..

[153] Francini-Pesenti F., Spinella P., Calò L.A. (2019). Potential role of phytochemicals in metabolic syndrome prevention and therapy. Diabetes Metab. Syndr. Obes. Targets Ther..

[154] Chen M.-L., Yi L., Zhang Y., Zhou X., Ran L., Yang J., Zhu J.-D., Zhang Q.-Y., Mi M.-T. (2016). Resveratrol attenuates trimethylamine-N-oxide (TMAO)-induced atherosclerosis by regulating TMAO synthesis and bile acid metabolism via remodeling of the gut microbiota. mBio.

[155] Zhao L., Zhang Q., Ma W., Tian F., Shen H., Zhou M. (2017). A combination of quercetin and resveratrol reduces obesity in high-fat diet-fed rats by modulation of gut microbiota. Food Funct..

[156] Liu W., Zhao S., Wang J., Shi J., Sun Y., Wang W., Ning G., Hong J., Liu R. (2017). Grape seed proanthocyanidin extract ameliorates inflammation and adiposity by modulating gut microbiota in high-fat diet mice. Mol. Nutr. Food Res..

[157] Papathanasopoulos A., Camilleri M. (2010). Dietary fiber supplements: Effects in obesity and metabolic syndrome and relationship to gastrointestinal functions. Gastroenterology.

[158] Hosseinpour-Niazi S., Mirmiran P., Sohrab G., Hosseini-Esfahani F., Azizi F. (2011). Inverse association between fruit, legume, and cereal fiber and the risk of metabolic syndrome: Tehran lipid and glucose study. Diabetes Res. Clin. Pract..

[159] Weickert M.O., Pfeiffer A.F. (2018). Impact of dietary fiber consumption on insulin resistance and the prevention of type 2 diabetes. J. Nutr..

[160] Galisteo M., Duarte J., Zarzuelo A. (2008). Effects of dietary fibers on disturbances clustered in the metabolic syndrome. J. Nutr. Biochem..

[161] Aleixandre A., Miguel M. (2008). Dietary fiber in the prevention and treatment of metabolic syndrome: A review. Crit. Rev. Food Sci. Nutr..

[162] Dewulf E.M., Cani P.D., Neyrinck A.M., Possemiers S., Van Holle A., Muccioli G.G., Deldicque L., Bindels L.B., Pachikian B.D., Sohet F.M. (2011). Inulin-type fructans with prebiotic properties counteract GPR43 overexpression and PPARγ-related adipogenesis in the white adipose tissue of high-fat diet-fed mice. J. Nutr. Biochem..

[163] Neyrinck A.M., Possemiers S., Druart C., Van de Wiele T., De Backer F., Cani P.D., Larondelle Y., Delzenne N.M. (2011). Prebiotic effects of wheat arabinoxylan related to the increase in bifidobacteria, Roseburia and Bacteroides/Prevotella in diet-induced obese mice. PLoS ONE.

[164] Farhangi M.A., Javid A.Z., Sarmadi B., Karimi P., Dehghan P. (2018). A randomized controlled trial on the efficacy of resistant dextrin, as functional food, in women with type 2 diabetes: Targeting the hypothalamic-pituitary-adrenal axis and immune system. Clin. Nutr..

[165] Sun Z., Sun X., Li J., Li Z., Hu Q., Li L., Hao X., Song M., Li C. (2020). Using probiotics for type 2 diabetes mellitus intervention: Advances, questions, and potential. Crit. Rev. Food Sci. Nutr..

[166] Lynch S.V., Pedersen O. (2016). The human intestinal microbiome in health and disease. N. Engl. J. Med..

[167] Cerdó T., García-Santos J.A., G Bermúdez M., Campoy C. (2019). The role of probiotics and prebiotics in the prevention and treatment of obesity. Nutrients.

[168] Plaza-Diaz J., Ruiz-Ojeda F.J., Gil-Campos M., Gil A. (2019). Mechanisms of action of probiotics. Adv. Nutr..

[169] Diehl A.M., Day C. (2017). Cause, pathogenesis, and treatment of nonalcoholic steatohepatitis. N. Engl. J. Med..

[170] Alcock J., Lin H.C. (2015). Fatty acids from diet and microbiota regulate energy metabolism. F1000Research.

[171] Lee J.Y., Sohn K.H., Rhee S.H., Hwang D. (2001). Saturated fatty acids, but not unsaturated fatty acids, induce the expression of cyclooxygenase-2 mediated through Toll-like receptor 4. J. Biol. Chem..

[172] Holzer R.G., Park E.-J., Li N., Tran H., Chen M., Choi C., Solinas G., Karin M. (2011). Saturated fatty acids induce c-Src clustering within membrane subdomains, leading to JNK activation. Cell.

[173] Bäckhed F., Ding H., Wang T., Hooper L.V., Koh G.Y., Nagy A., Semenkovich C.F., Gordon J.I. (2004). The gut microbiota as an environmental factor that regulates fat storage. Proc. Natl. Acad. Sci. USA.

[174] Ding S., Chi M.M., Scull B.P., Rigby R., Schwerbrock N.M., Magness S., Jobin C., Lund P.K. (2010). High-fat diet: Bacteria interactions promote intestinal inflammation which precedes and correlates with obesity and insulin resistance in mouse. PLoS ONE.

[175] Devkota S., Wang Y., Musch M.W., Leone V., Fehlner-Peach H., Nadimpalli A., Antonopoulos D.A., Jabri B., Chang E.B. (2012). Dietary-fat-induced taurocholic acid promotes pathobiont expansion and colitis in Il10−/− mice. Nature.

[176] Kim K.-A., Gu W., Lee I.-A., Joh E.-H., Kim D.-H. (2012). High fat diet-induced gut microbiota exacerbates inflammation and obesity in mice via the TLR4 signaling pathway. PLoS ONE.

[177] Laugerette F., Furet J.-P., Debard C., Daira P., Loizon E., Géloën A., Soulage C.O., Simonet C., Lefils-Lacourtablaise J., Bernoud-Hubac N. (2012). Oil composition of high-fat diet affects metabolic inflammation differently in connection with endotoxin receptors in mice. Am. J. Physiol. Endocrinol. Metab..

[178] Jakobsdottir G., Xu J., Molin G., Ahrne S., Nyman M. (2013). High-fat diet reduces the formation of butyrate, but increases succinate, inflammation, liver fat and cholesterol in rats, while dietary fibre counteracts these effects. PLoS ONE.

[179] Ghosh S., DeCoffe D., Brown K., Rajendiran E., Estaki M., Dai C., Yip A., Gibson D.L. (2013). Fish oil attenuates omega-6 polyunsaturated fatty acid-induced dysbiosis and infectious colitis but impairs LPS dephosphorylation activity causing sepsis. PLoS ONE.

[180] Hildebrandt M.A., Hoffmann C., Sherrill-Mix S.A., Keilbaugh S.A., Hamady M., Chen Y.Y., Knight R., Ahima R.S., Bushman F., Wu G.D. (2009). High-fat diet determines the composition of the murine gut microbiome independently of obesity. Gastroenterology.

[181] Zeng H., Liu J., Jackson M.I., Zhao F.-Q., Yan L., Combs G.F. (2013). Fatty liver accompanies an increase in lactobacillus species in the hind gut of C57BL/6 mice fed a high-fat diet. J. Nutr..

[182] Huang E.Y., Leone V.A., Devkota S., Wang Y., Brady M.J., Chang E.B. (2013). Composition of dietary fat source shapes gut microbiota architecture and alters host inflammatory mediators in mouse adipose tissue. J. Parenter. Enter. Nutr..

[183] Kaliannan K., Wang B., Li X.-Y., Bhan A.K., Kang J.X. (2016). Omega-3 fatty acids prevent early-life antibiotic exposure-induced gut microbiota dysbiosis and later-life obesity. Int. J. Obes..

[184] Del Rio D., Rodriguez-Mateos A., Spencer J.P., Tognolini M., Borges G., Crozier A. (2013). Dietary (poly) phenolics in human health: Structures, bioavailability, and evidence of protective effects against chronic diseases. Antioxid. Redox Signal..

[185] Tomás-Barberán F.A., Selma M.V., Espín J.C. (2016). Interactions of gut microbiota with dietary polyphenols and consequences to human health. Curr. Opin. Clin. Nutr. Metab. Care.

[186] Zhang Y., Bobe G., Revel J.S., Rodrigues R.R., Sharpton T.J., Fantacone M.L., Raslan K., Miranda C.L., Lowry M.B., Blakemore P.R. (2020). Improvements in metabolic syndrome by xanthohumol derivatives are linked to altered gut microbiota and bile acid metabolism. Mol. Nutr. Food Res..

[187] Anhê F.F., Roy D., Pilon G., Dudonné S., Matamoros S., Varin T.V., Garofalo C., Moine Q., Desjardins Y., Levy E. (2015). A polyphenol-rich cranberry extract protects from diet-induced obesity, insulin resistance and intestinal inflammation in association with increased Akkermansia spp. population in the gut microbiota of mice. Gut.

[188] Szilagyi A. (2019). Relationship (s) between obesity and inflammatory bowel diseases: Possible intertwined pathogenic mechanisms. Clin. J. Gastroenterol..

